# Interleukin-15: a possible link between anorexia nervosa and schizophrenia

**DOI:** 10.1192/j.eurpsy.2024.1428

**Published:** 2024-08-27

**Authors:** P. Lederer, A. Mihaljević-Peleš, I. Begovac, M. Tripković

**Affiliations:** Department of Psychiatry and Psychological Medicine, University hospital Centre Zagreb, Zagreb, Croatia

## Abstract

**Introduction:**

Interleukin-15 is a cytokine that induces or enhances differentiation, maintenance, or activation of several T-cell subsets (including NK, NKT, Th17, Treg, and CD8+ memory cells) and also plays an important role in regulating visceral (intra-abdominal or interstitial) fat breakdown and myofibrillar protein synthesis (hypertrophy). It is also involved in modulating serotonergic activity in the brain by modulating the transmission of GABA and serotonin, which may be the basis for mood and memory disorders, as well as activity levels, sleep, and thermoregulation. Both anorexia nervosa (AN) and schizophrenia (SCH) represent two distinct and serious psychiatric disorders in which some of these symptoms may overlap and where neuroinflammation plays an important role which is yet to be precisely determined.

**Objectives:**

This article summarizes recent findings and highlights interleukin-15 as a possible link between anorexia nervosa and schizophrenia.

**Methods:**

A review of the current literature in the field of psychoneuroimmunology.

**Results:**

In recent years, research has shown elevated levels of IL -15 in the serum of patients suffering from anorexia nervosa and schizophrenia.It is also interesting to note that IL -15 has structural similarities to IL -2, which previous studies have also shown to be elevated in patients with schizophrenia.

**Conclusions:**

These associations, so far suggesting an important role of inflammation and its mediators, need further investigation in light of possible genetic overlap between anorexia nervosa and schizophrenia identified in genome-wide association studies (GWAS).

**Disclosure of Interest:**

None Declared

